# Central Venous Pressure and Impaired Renal Function in Children and Young Adults With Cardiovascular Disease

**DOI:** 10.1016/j.jacadv.2024.100995

**Published:** 2024-05-21

**Authors:** Jillian Olsen, Hari Tunuguntla, Alexander Alali, Swati Choudhry, Kyle D. Hope, Kriti Puri, Joseph A. Spinner, Ayse Akcan-Arikan, Jack F. Price

**Affiliations:** a Department of Pediatrics, Division of Cardiology, Baylor College of Medicine/Texas Children's Hospital, Houston, Texas, USA; b Department of Pediatrics, Division of Critical Care Medicine, Baylor College of Medicine/Texas Children's Hospital, Houston, Texas, USA; c Division of Critical Care Medicine

**Keywords:** cardiorenal syndrome, central venous pressure, kidney injury, renal dysfunction

## Abstract

**Background:**

Traditionally, low cardiac output has been considered the primary hemodynamic driver of renal function and injury. Adult data suggest that central venous pressure (CVP) is a more important factor.

**Objectives:**

The authors hypothesized that in children with cardiovascular disease, higher CVP predicts lower estimated glomerular filtration rate (eGFR) and worsening renal function (WRF).

**Methods:**

We performed a single-center cohort study of patients aged 3 months to 21 years with biventricular circulation undergoing cardiac catheterization. Pearson’s correlation and linear and Cox regression analyses were performed to determine associations with eGFR at the time of catheterization and WFR within 180 days after catheterization.

**Results:**

312 patients had median age 7.9 years (IQR: 2.3 to 14.5 years), median eGFR 97 mL/min/1.73 m^2^ (IQR: 81-118 mL/min/1.73 m^2^), median CVP 7 mm Hg (IQR: 5-9 mm Hg), and median cardiac index 3.7 mL/min/m^2^ (IQR: 2.9-4.6 mL/min/m^2^). Nearly half (48%) were transplant recipients. In multivariable analysis, CVP was independently associated with eGFR (β = −2.65; 95% CI: −4.02, −1.28; *P* < 0.001), as was being a transplant recipient (β = −10.20; 95% CI: −17.74, −2.65; *P* = 0.008), while cardiac index was not. Fifty-one patients (16%) developed WRF. In a proportional hazards model adjusting for cardiac index, only higher CVP (HR: 1.10; 95% CI: 1.04-1.17; *P* = 0.002) and greater contrast volume by weight (HR: 1.05; 95% CI: 1.01-1.10; *P* = 0.021) predicted WRF. CVP ≥7 mm Hg likewise predicted WRF (HR: 2.57; 95% CI: 1.29-5.12; *P* = 0.007).

**Conclusions:**

Among children with a spectrum of cardiovascular disease, higher CVP is associated with lower eGFR and development of WRF, independent of cardiac index.

Renal dysfunction is a common comorbidity and risk factor for adverse outcomes including death and cardiac transplantation in both adults and children with heart disease.^1^, ^2^, ^3^, ^4^ Despite its important prognostic value, the pathophysiology, prevention, and management of the cardiorenal syndrome remain incompletely understood, especially in children. Renal dysfunction with coexisting cardiovascular disease is often attributed to a deficiency in antegrade perfusion, or “prerenal” failure. Traditional teaching assumes that reduced cardiac output and subsequent neurohormonal activation lead to increased serum creatinine and declining renal performance. However, this explanation ignores the contribution of venous congestion to renal function. In adults with cardiac disease, increased central venous pressure (CVP) is independently associated with impaired renal function and adverse outcomes^5^ and appears to be the main hemodynamic driver of worsening renal function (WRF) over time.^6^ Proposed mechanisms for this phenomenon include compression of the distal tubules by distended venules effectively causing an obstructive uropathy, as well as the sympathetic activation and angiotensin cascade initiated by increased renal capsular interstitial pressure.^7^, ^8^, ^9^, ^10^, ^11^ Unlike in adults, in children, the relationship between CVP and renal function has not been well described. We have previously shown that in patients who have undergone the Fontan palliation for single ventricle physiology, higher CVP is associated with reduced estimated glomerular filtration rate (eGFR).^12^ Similarly, in a cohort of very low birth weight infants without cardiac disease, higher CVP was associated with increased serum creatinine and diminished urine output.^13^ Across a broad group of children with cardiovascular disease and biventricular physiology, though, the relationship between CVP and renal function has not been established. Furthermore, the prognostic value of venous congestion for predicting WRF in children has never been examined. Thus, we sought to test the hypothesis that, in children with a spectrum of cardiovascular disease, CVP correlates with eGFR and predicts WRF.

## Methods

### Study design

We performed a retrospective cohort study of consecutive patients aged 3 months to 21 years who underwent cardiac catheterization with hemodynamic assessment for any indication at our institution between December 2008 and January 2018. For patients who had multiple catheterizations performed during the study period, data from only their first procedure were included. For cardiac transplant patients, data were collected from the first catheterization at least 1 year after transplant to limit confounding related to the immediate pre- and post-transplant hemodynamics and induction immunosuppression. Children with intracardiac shunting or pre-existing chronic kidney disease and those on inotropes, vasopressors, or mechanical circulatory support were excluded. We likewise excluded any patients who had not undergone echocardiography within 90 days preceding the catheterization and those without a serum creatinine concentration measured within 72 hours prior to the catheterization. The study design was approved by the Institutional Review Board of Baylor College of Medicine, and parental and patient consent was waived.

Demographic characteristics, laboratory data, echocardiographic data, medications patients were taking at the time of catheterization, and hemodynamic measurements were collected. Laboratory data included serum creatinine, hemoglobin, serum sodium, and B-type natriuretic peptide (BNP) at the time of catheterization. Qualitative and quantitative estimated ventricular systolic function and estimated degree of tricuspid regurgitation were obtained from each patient’s most recent echocardiogram prior to catheterization.

Standard hemodynamic measurements obtained during catheterization included systemic blood pressure, right atrial pressure (CVP), pulmonary capillary wedge pressure (PCWP), mean pulmonary arterial pressure (PAP), and arterial to venous oxygen saturation (A-VO_2_) difference. Cardiac index (L/min/m^2^) was calculated using the Fick method with an assumed oxygen consumption based on age, sex, and resting or average heart rate during the catheterization. The high superior vena cava saturation was included in our calculations when available; otherwise, the right atrial or pulmonary artery saturation was used. These measurements were usually acquired while the patient was intubated and sedated. The systolic blood pressure z-score was derived from expected values obtained from published age-, sex-, and height-based norms.^14^

The primary independent variables of interest were eGFR and WRF, defined as an increase in creatinine of at least 0.3 mg/dL within 180 days following catheterization. Previous studies have identified this degree of creatinine change as clinically significant in both children and adults.^1^^,^^15^, ^16^, ^17^ The modified bedside Schwartz equation (0.413 · height in centimeters/serum creatinine in mg/dL) was used to calculate eGFR at the time of catheterization.^18^

### Statistical analysis

Baseline characteristics are expressed as median (IQR) for continuous data and as percentages for categorical data. A receiver operating characteristic curve was generated to determine the most discriminating value of CVP in predicting WRF. Using the derived cutoff based on a maximum product of sensitivity and specificity, CVP was analyzed as both a continuous and dichotomous variable when testing for association with WRF. The *t*-test for independent samples was used to detect a difference in mean eGFR between groups. The Kruskal-Wallis test was used to test the difference in mean eGFR within groups. Linear regression methods were used to explore univariable and multivariable associations with eGFR. Polynomial regression analysis was performed to explore nonlinearity between multiple clinical variables and eGFR. Variables analyzed included age, sex, transplant status, medications, CVP, cardiac index, A-VO_2_ difference, PCWP, PAP, systolic blood pressure z-score, the presence of at least moderate ventricular systolic dysfunction, and at least qualitatively moderate tricuspid regurgitation on echocardiogram. Cox proportional hazard models were created using time dependent covariates to identify associations with development of WRF by 180 days. Covariates that had a *P* value <0.10 on univariable analysis as well as cardiac index as a variable of particular interest were included in a multivariable Cox regression. In cases of with collinearity or overlapping implications (eg, cardiac index and A-VO_2_ difference as measures of cardiac output), input variables were limited in order to avoid overfitting. A Kaplan-Meier curve was constructed for CVP and WRF using the log-rank test. A *P* value <0.05 was considered statistically significant throughout the analysis. Statistical analysis was performed using IBM SPSS Statistics, version 28.0 (IBM Corp) and Stata/MP, version 17.0 (StatCorp).

## Results

A total of 312 patients were included (Table 1), most of whom were school-aged children with a median age of 7.9 years (IQR: 2.3-14.5 years) and a slight male predominance (59%). Due to the exclusion of patients with intracardiac shunting and single ventricle physiology, nearly half of our patients were heart transplant recipients (48%), many of whom were undergoing routine screening right ventricular biopsies. All heart transplant patients were receiving a calcineurin inhibitor at the time of catheterization. Of the congenital heart disease group (31%), about half were undergoing angioplasty and/or stent placement (n = 45; 46%), a third were undergoing hemodynamic studies (n = 32; 33%), and the remainder were being treated with balloon valvuloplasty or transcatheter valve placement (n = 20; 21%). A minority of patients had cardiomyopathy (n = 34; 11%) or primary pulmonary hypertension (n = 28; 9%).

**Table 1:** Baseline Patient Characteristics at the Time of Catheterization (N = 312)

Age (y)	7.9 (2.3-14.5)
Male	183 (59)
Weight (kg)	22.6 (11.3-52.5)
Height (cm)	121.5 (84-154)
Diagnosis	
Cardiac transplant	150 (48)
Congenital heart disease	97 (31)
Cardiomyopathy	34 (11)
Pulmonary hypertension	28 (9)
Other	3 (1)
Indication for catheterization	
Transplant surveillance	143 (46)
Hemodynamics	52 (17)
Angioplasty/stent placement	48 (15)
Pulmonary hypertension study	28 (9)
Device implantation (eg, defibrillator)	16 (5)
Balloon valvuloplasty	12 (4)
Diagnostic biopsy	7 (2)
Other	6 (2)
Echocardiogram	
Left ventricular ejection fraction (%) (n = 214)	64 (58-67)
At least moderate left ventricular systolic dysfunction (n = 299)	29 (9)
At least moderate right ventricular systolic dysfunction (n = 299)	31 (10)
At least moderate tricuspid regurgitation (n = 299)	30 (10)
Catheterization data	
Intravenous contrast load (mL/kg) (n = 309)	0.61 (0.26-2.3)
Central venous pressure (mm Hg) (n = 312)	7 (5-9)
Pulmonary capillary wedge pressure (mm Hg) (n = 300)	10 (8-13)
Mean pulmonary artery pressure (mm Hg) (n = 306)	17 (15-24)
Cardiac index (L/min/m^2^) (n = 307)	3.7 (2.9-4.6)
Arteriovenous oxygen saturation difference (%) (n = 311)	26 (22-32)
Systolic blood pressure (mm Hg) (n = 305)	84 (76-94)
Diastolic blood pressure (mm Hg)	49 (42-55)
Systolic blood pressure Z-score, (n = 305)	−1.1 (−1.9 to −0.4)
Diastolic blood pressure Z-score (n = 305)	−0.5 (−1.0 to 0.0)
Laboratory	
B-type natriuretic peptide (pg/mL) (n = 195)	75 (40-285)
eGFR (mL/min/1.73m^2^)	97 (81-118)
Medications	
ACE inhibitor or angiotensin receptor blocker	92 (29)
Diuretic	73 (23)
Beta-blocker	59 (19)
Pulmonary vasodilator	12 (4)
Aldosterone antagonist	9 (3)

Values are median (IQR) or n (%).

ACE = angiotensin-converting enzyme; eGFR = estimated glomerular filtration rate.

Most patients had hemodynamic values within a normal or near-normal range: median CVP was 7 mm Hg (IQR: 5-9 mm Hg), median PCWP was 10 mm Hg (IQR: 8-13 mm Hg), and median PAP was 17 mm Hg (IQR: 15-24 mm Hg). Calculated estimates of cardiac output were also predominantly within normal range and included a median cardiac index of 3.7 L/min/m^2^ (IQR: 2.9-4.6 L/min/m^2^) and median A-VO_2_ difference of 26% (IQR: 22%-32%). Only 63% of patients had a BNP drawn within 3 days prior to catheterization with a median of 75 pg/mL (IQR: 40-285 pg/mL), so BNP was not included in the analyses. All but 13 patients had relevant echocardiographic data available on ventricular and tricuspid valve function, and the majority had well-preserved cardiac function, with just under 10% having at least moderate right or left ventricular systolic dysfunction. The proportion of moderate or greater tricuspid regurgitation was similar (10%). Very few patients were taking pulmonary vasodilators or aldosterone antagonists; one-fifth were on beta-blockers (19%), one-quarter were on diuretics (23%), and nearly one-third were taking an angiotensin-converting enzyme inhibitor or angiotensin receptor blocker (29%) at the time of catheterization.

### Analysis of estimated glomerular filtration rate

**Figure 1:**
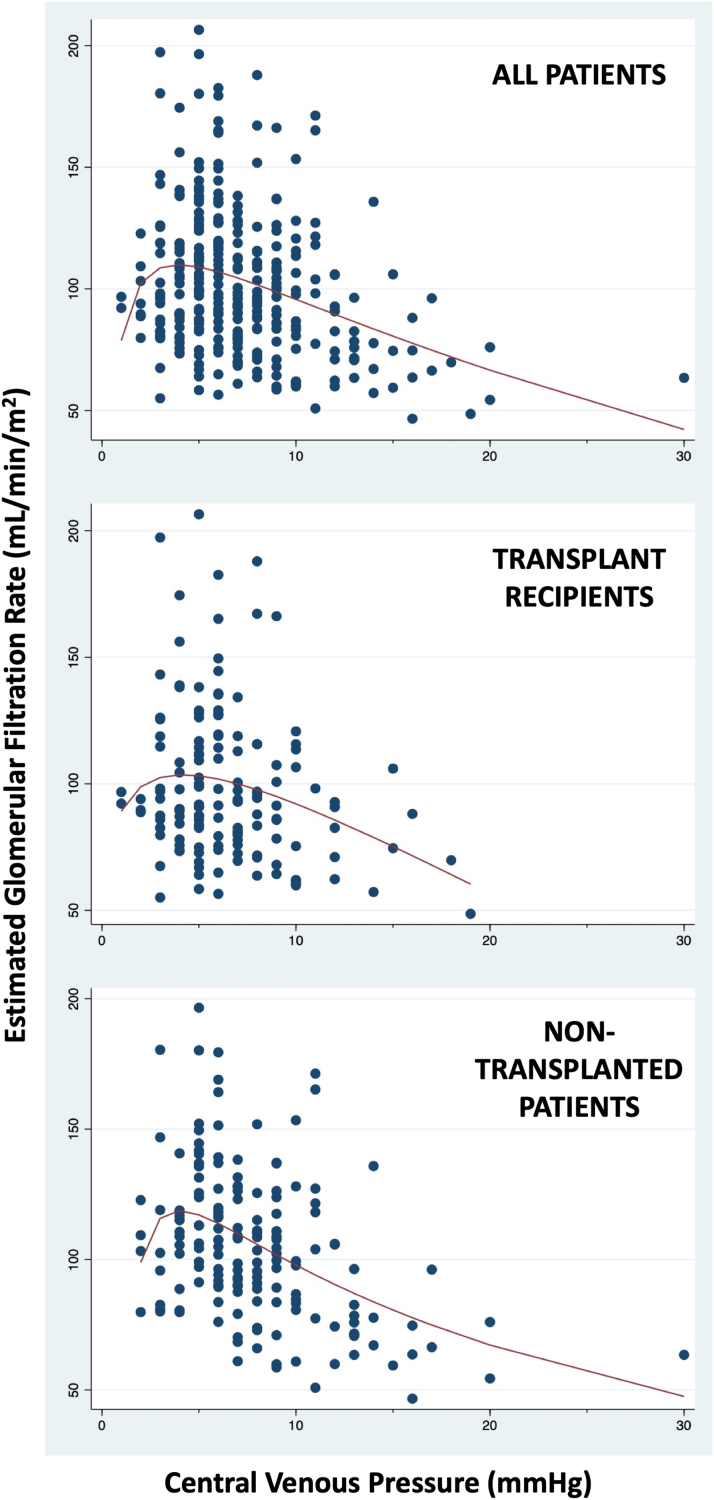
**Curvilinear Relationship Between Central Venous Pressure and Estimated Glomerular Filtration Rate** Estimated glomerular filtration rate (eGFR) increases at low central venous pressure and decreases at high central venous pressure. The relationship holds true when transplanted and non-transplanted patients are examined separately. The best fit curve by second degree fractional polynomial regression pictured above for all patients is defined by eGFR = 78.9 + 45.2·ln(CVP) − 16.5·(ln(CVP)^2^ (*P* < 0.001). CVP = central venous pressure.

The median eGFR at the time of catheterization was 97 mL/min/1.73 m^2^ (IQR: 81-118 mL/min/1.73 m^2^). Only 13 patients (4%) had an eGFR <60 mL/min/1.73 m^2^. Figure 1 demonstrates a modest and curvilinear relationship between CVP and eGFR as obtained by fractional polynomial modeling; because of the large proportion of transplant recipients in our population, these patients are presented separately in addition to the whole cohort. For CVP ≤6 mm Hg, the correlation with eGFR was direct (r = 0.16, *P* = 0.050) and for CVP >6 mm Hg, the relationship was indirect (r = −0.32, *P* = 0.001), with an overall negative correlation (r = −0.29, *P* < 0.001). Even for patients with “normal range” CVPs, the IQR of 5 to 9 mm Hg, the negative correlation with eGFR persists (r = −0.18, *P* = 0.013), and when the outlier with a CVP of 30 mm Hg was removed from the analysis, the correlation of CVP to eGFR changed little. The relationship was similar when transplant patients were examined alone (r = −0.22, *P* = 0.008 for transplant recipients, r = −0.40, *P* < 0.001 for non-transplanted patients). Subgroup analysis based on cardiac index confirmed the inverse correlation between eGFR and CVP (r = −0.23, *P* < 0.001) among patients with normal cardiac index (>3.0 L/min/m^2^).

In univariable linear regression models, patient age, sex, A-VO_2_ difference, the presence of at least moderate tricuspid regurgitation, systolic blood pressure z-score, and medications were not associated with eGFR (Supplemental Table). Cardiac index was likewise not associated with eGFR on linear regression, though curvilinear regression analysis revealed a weak correlation (r = 0.18, *P* = 0.002). Subgroup analysis revealed that patients with abnormally low cardiac index (≤2.3 mL/min/m^2^, N = 36) had a lower mean eGFR than the remainder of the cohort (88 vs 99 mL/min/m^2^, *P* < 0.001). An additional subanalysis was performed to identify associations between eGFR and catheterization indication. We tested the hypothesis that mean eGFR was the same across indications for catheterization and found that patients undergoing transplant surveillance or a pulmonary hypertension study had the lowest eGFR (*P* = 0.029).

On multivariable analysis, CVP was independently associated with eGFR (β = −2.65; 95% CI: −4.02 to −1.28; *P* < 0.001) but cardiac index was not (β = −2.22; 95% CI: -6.07 to 1.64; *P* = 0.259). We also identified a strong association between eGFR and cardiac transplant status (β = −10.20; 95% CI: −17.74 to −2.65; *P* = 0.008). All transplant patients were receiving potentially nephrotoxic medications. Multivariable analysis results were nearly identical when transplanted and non-transplanted patients were each analyzed separately, with CVP as the only variable independently associated with eGFR (β = −3.26; 95% CI: −5.81 to −0.71; *P* = 0.013 and β = −2.35; 95% CI: −3.93 to −0.77; *P* = 0.004, respectively). Notably, the adjusted r^2^ of the overall multivariable model was only 0.13, indicating that hemodynamic measurements account for only a small proportion of the variability of eGFR in this cohort.

### Analysis of worsening renal function

WRF occurred in 51 children (16%) within 180 days after catheterization. The median lowest eGFR among those with WRF during the 180 day post-catheterization period was 39 mL/min/m^2^ (IQR: 2-51 mL/min/m^2^) and the median maximum percent decrease in eGFR was 64% (IQR: 41%-75%). The receiver operating characteristic curve analysis identified a CVP of ≥7 mm Hg as most discerning in predicting WRF, with half of patients (50%) meeting this cutoff (c-statistic = 0.68), so CVP was subsequently investigated as both a continuous and dichotomous variable above and below 7 mm Hg.

Univariable Cox regression analysis (Table 2) showed that just as CVP was associated with lower eGFR, higher CVP was also an important risk factor for WRF. In unadjusted analyses, we found significant associations between WRF and CVP as a continuous variable, CVP ≥7 mm Hg, cardiac index, A-VO_2_ difference, at least moderate tricuspid regurgitation, cardiac transplant status, PCWP, and mean PAP. Both CVP and cardiac index were associated with lowest eGFR during the 180 day post-catheterization period (β = −1.19; 95% CI: −2.35 to −0.045; *P* = 0.042 and β = 4.66; 95% CI: 0.48-8.26; *P* = 0.029). CVP and cardiac index were likewise both associated with a greater maximum increase in creatinine in the first year after catheterization (β = 0.05; 95% CI: 0.03-0.07; *P* = 0.070 and β = −0.08; 95% CI: −0.14 to −0.01; *P* = 0.020). Patient age, sex, use of cardiac medications, ventricular dysfunction, and systolic blood pressure z-score were not associated with WRF in univariable analyses.

**Table 2:** Cox Regression for Predictors of Worsening Renal Function Within 180 Days of Catheterization

	Unadjusted Analysis HR (95% CI)	*P* Value	Adjusted Analysis HR (95% CI)	*P* Value
Age	1.02 (0.98-1.07)	0.332		
Male	1.42 (0.80-2.55)	0.234		
Transplant recipient	0.30 (0.12-0.75)	0.010	1.22 (0.61-2.44)	0.572
Contrast volume (mL/kg)	1.04 (1.00-1.09)	0.052	1.05 (1.01-1.10)	0.021
CVP	1.13 (1.08-1.18)	<0.001	1.10 (1.04-1.17)	0.002
CVP ≥7 mm Hg^a^	3.30 (1.76-6.19)	<0.001	2.57 (1.29-5.12)	0.007
Cardiac index	0.67 (0.52-0.86)	0.002	0.76 (0.57-1.01)	0.062
A-VO_2_ difference	1.07 (1.04-1.07)	<0.001		
LV dysfunction	1.77 (0.79-3.95)	0.163		
RV dysfunction	1.96 (0.92-4.19)	0.082		
At least moderate TR	2.68 (1.34-5.39)	0.006	1.52 (0.67-3.41)	0.315
PCWP	1.09 (1.04-1.14)	<0.001		
Mean PAP	1.02 (1.00-1.04)	0.033		
Systolic BP z-score	1.01 (0.95-1.07)	0.758		
Diuretic	1.77 (0.99-3.17)	0.054	1.44 (0.77-2.68)	0.258
ACEi or ARB	0.63 (0.32-1.22)	0.175		
Beta-blocker	1.19 (0.61-2.32)	0.608		

ACEi = angiotensin converting enzyme inhibitor; ARB = angiotensin receptor blocker; A-VO_2_ = arterial-venous oxygen saturation; BP = blood pressure; CVP = central venous pressure; LV = left ventricular; PAP = pulmonary artery pressure; PCWP = pulmonary capillary wedge pressure; RV = right ventricular; TR = tricuspid regurgitation.

a When replacing the continuous variable CVP.

Although intravenous contrast volume and use of diuretics did not reach statistical significance in univariable analyses, we entered both into a multivariable proportional hazards model along with CVP, transplant status, cardiac index, and tricuspid regurgitation to test for association with WRF. Only greater contrast volume by weight (HR: 1.05; 95% CI: 1.01-1.10; *P* = 0.021) and higher CVP (HR: 1.10; 95% CI: 1.04-1.17; *P* = 0.002) were associated with declining renal function over time. For every 1 mm Hg increase in CVP, the risk of WRF increased by 10%. When CVP ≥7 mm Hg replaced the continuous variable CVP in the model, it too was independently associated with WRF (HR: 2.57; 95% CI: 1.29-5.12; *P* = 0.007), more than doubling the risk of kidney injury even when adjusting for cardiac index. For CVP ≥10 mm Hg, the HR for WRF increased to 3.37 (95% CI: 1.82-6.22; *P* < 0.001). The univariable association between cardiac index and WRF was lost in the multivariable analysis (HR: 0.76; 95% CI: 0.57-1.01; *P* = 0.062).

In a subgroup analysis of patients with relatively low eGFR (<90 mL/min/m^2^), those who developed WRF had a higher CVP at the time of catheterization (10 mm Hg [IQR: 7-13 mm Hg] vs 7 mm Hg [IQR: 5-9 mm Hg], *P* = 0.018), but no difference in cardiac index compared to those who did not develop WRF (3.6 mL/min/m^2^ (IQR: 2.1-4.4 mL/min/m^2^) vs 3.9 mL/min/m^2^ (IQR: 2.9-4.9 mL/min/m^2^), *P* = 0.089). For non-transplanted patients with low eGFR, the result was the same, with a higher CVP in those who developed WRF (12.5 mm Hg [IQR: 10-16 mm Hg] vs 8 mm Hg [IQR: 6-12 mm Hg], *P* = 0.013). Transplanted patients with low eGFR showed a similar but nonsignificant trend in CVP (6.5 mm Hg [IQR: 5-10 mm Hg] vs 6 mm Hg [IQR: 4-8 mm Hg] in those who did and did not develop WRF, respectively). A Kaplan-Meier curve is provided for visualization of the association between CVP and freedom from WRF over time (Central Illustration).

**Central Illustration:**
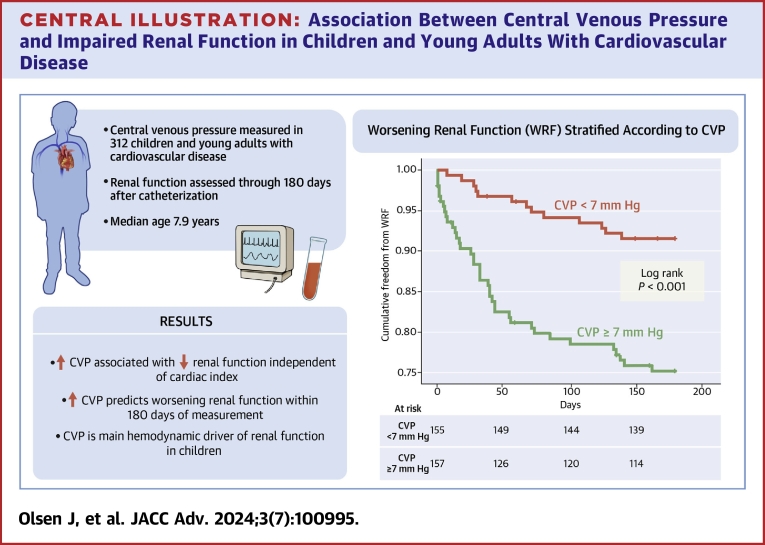
**Association Between Central Venous Pressure and Impaired Renal Function in Children and Young Adults With Cardiovascular Disease** Increased CVP is associated with impaired and worsening renal function in children with a broad spectrum of cardiovascular disease, even when adjusting for cardiac index. CVP is the main hemodynamic driver of renal function in children. A Kaplan-Meier curve demonstrates the elevated risk conferred by a CVP ≥7 mm Hg in developing worsening renal function as defined by an increase in serum creatinine of at least 0.3 mg/dL within 180 days following catheterization in children age 3 months to 21 years. CVP = central venous pressure; WRF = worsening renal function.

## Discussion

The primary findings of this study are that higher CVP is associated with lower eGFR in children with a variety of cardiovascular disorders, even when adjusting for markers of cardiac performance and oxygen delivery and that increased CVP is the strongest hemodynamic predictor of WRF over time in this cohort of children.

### Central venous pressure and estimated glomerular filtration rate

In the present study, we observed that the relationship between CVP and kidney function is curvilinear, with eGFR increasing slightly as CVP rises in the low single digits and decreasing more acutely as CVP increases beyond 6 mm Hg. Along a spectrum of increasing venous pressures, these changes in renal function may reflect improved renal blood flow due to increased circulating blood volume up to an optimal point followed by a detrimental impact of venous pressure beyond that point. The lower eGFR that we identified at the lowest CVPs may reflect the transient acute kidney injury that is often observed when aggressively decongesting children with heart failure. Our findings are strikingly similar to those observed by Damman et al in adults, including the same inflection point at 6 mm Hg.^5^ These data confirm, for the first time in children, that the relationship between renal function and CVP is biphasic, frequently reduced at lowest and highest venous pressures and usually optimal at “normal” venous pressures. Whereas volume resuscitation or decreased diuresis is often a response to acute kidney injury, our findings leave room for doubt that this is always the most appropriate approach for a patient who may already have a “normal” CVP.

Our findings in a pediatric cohort are similar to those in adult studies in which higher CVP predicted renal function and injury.^5^, ^6^, ^7^^,^^19^, ^20^, ^21^ In the largest of these, Damman et al identified a strong and independent association between CVP and lower eGFR in more than 2,500 adults undergoing catheterization for a broad range of indications.^5^ In a separate study of patients with acute heart failure and low systolic blood pressure, Uthoff et al showed that CVP was significantly associated with lower eGFR, similar to our findings in this pediatric cohort with and without heart failure.^21^ To our knowledge, though, the current study is the first to demonstrate the relationship between CVP and eGFR in a broader pediatric population, most with preserved systolic function. Results were consistent across patients with and without a transplanted heart, suggesting that these findings are not attributable to nephrotoxic immunosuppression or a pretransplant low cardiac output state.

### Central venous pressure and worsening renal function

This also represents the first work in children to report the prognostic importance of CVP for predicting subsequent decline in renal function. Among adults hospitalized with decompensated heart failure, Mullens et al showed that a higher CVP on admission was an independent risk factor for WRF, but the same has not been previously shown in children.^6^ Our data also demonstrate the strong predictive value of CVP as both a continuous and dichotomous variable at an optimal cutoff of 7 mm Hg in the development of WRF. Children with CVP ≥7 mm Hg were more than twice as likely to develop WRF within 180 days of hemodynamic assessment compared to their peers with a lower CVP, even when adjusting for cardiac index. This cutoff must be interpreted with the understanding that hemodynamic data were collected under anesthesia, so a patient with a value of 7 mm Hg on catheterization may have a higher CVP awake.

The association between high CVP and WRF may be inferred from other studies demonstrating that volume-overloaded heart failure and cirrhosis patients commonly develop at least transient kidney injury in both adult and pediatric cohorts.^1^^,^^2^^,^^15^^,^^22^ Other factors that raise CVP, including tricuspid regurgitation, right heart failure, and pulmonary hypertension, have also been associated with renal dysfunction and mortality.^23^, ^24^, ^25^ Even in noncardiac disease states such as sepsis, increased CVP is associated with worse outcomes.^26^ The concept of venous hypertension leading to renal impairment is not new. In 1931, Winton demonstrated that an acute increase in renal vein pressure in dogs was followed by decreased renal blood flow and urine production and increased urea secretion.^8^ He speculated that increased pressure in the vein was transmitted to the tubules, slowing the secretion of urine in a manner similar to increased pressure in the ureter. Proposed mechanisms for “congestive kidney failure” describe intrarenal venous hypertension causing increased renal capsular interstitial pressure and a systemic neurohormonal response activating the angiotensin cascade and sympathetic nervous system.^7^^,^^27^, ^28^, ^29^ This leads to reduced glomerular filtration and enhanced sodium retention, thereby expanding plasma volume and further increasing CVP in patients with edematous states such as heart failure and cirrhosis.^30^ Based on these potential mechanisms, hemodynamic perturbations such as tricuspid regurgitation and right ventricular dysfunction would impact renal dysfunction only if they lead to a change in CVP that can be sensed by the glomerular capsule or distal tubules. The compliance and distensibility of the right atrium thus may be protective and explain why tricuspid regurgitation and right ventricular dysfunction were not associated with lower eGFR in multivariate analysis.

### Cardiac index, blood pressure, and renal function

In our cohort of patients with relatively preserved systolic function and normal hemodynamic parameters, cardiac index was not associated with baseline renal function in multivariable models, nor was it predictive of WRF 180 days after hemodynamic assessment. Again, these findings reiterate the conclusions of adult studies.^6^ Data from the ESCAPE (Evaluation Study of Congestive Heart Failure and Pulmonary Artery Catheterization Effectiveness) trial challenged the belief that cardiac index is a predominant driver of renal function, revealing no association between cardiac index and renal function or between a change in cardiac index and change in renal function.^19^ It is important to point out, however, that the patients in our study with the lowest cardiac index (<2.3 mL/min/m^2^) did have reduced eGFR relative to the rest of the cohort. Even so, cardiac index was not associated with WRF at 180 days, including among those with the lowest eGFR. It may be that the greatest influence of cardiac index on kidney function occurs in patients with heart failure, at least among children. In another pediatric cohort with end-stage heart failure who underwent hemodynamic assessment prior to transplant, cardiac index was associated with abnormally low eGFR at the time of catheterization.^31^ Heart failure as a clinical syndrome is a known risk for developing WRF, even in children; however, the relationship of venous congestion and WRF in these patients has never been examined and is worthy of study.^1^

Although cardiac index does not appear to contribute greatly to renal performance, at least not in the relatively normal range of values represented in our cohort, systemic arterial blood pressure and renal blood flow may play important roles. Dupont et al demonstrated that in adults hospitalized with acute decompensated heart failure receiving pulmonary artery catheter-guided treatment, lower mean arterial blood pressure correlated weakly with WRF, to a greater extent than did changes in CVP.^20^ Renal blood flow is influenced by both arterial and venous blood pressures as well as vascular resistance and intra-abdominal pressure. In a cohort of adults with advanced pulmonary hypertension in whom renal blood flow was measured, venous pressure and GFR were independently associated with renal blood flow. The lowest eGFR was observed in patients with high CVP and low renal blood flow while those patients with high CVP and high renal blood flow had relatively normal kidney function.^24^ In other words, in that adult cohort, the relative contribution of CVP to eGFR appeared to be limited to patients with low renal blood flow. We did not find a relationship between systolic blood pressure z-score measured at a single point in time and CVP or WRF in our analysis. Serial measures of arterial blood pressure were not performed and thus dynamic changes in blood pressure could not be tested for an association with renal function.

### Additional risk factors for renal dysfunction

Tubular injury secondary to nephrotoxic medications or intravenous contrast may exacerbate the influence of high venous pressure on kidney function. This may help explain our finding that cardiac transplant status was strongly and independently linked to reduced eGFR, though we detected no increased risk of developing WRF in those children. All transplant patients had been receiving calcineurin inhibitors for at least 1 year and were thus likely to be at drug steady state. Although exposure to higher contrast load by weight predicted WRF in our study, CVP remained associated with WRF when contrast load was included in adjusted analysis.

### Study limitations

Several important limitations should be considered when interpreting our findings. First, while our patient population consists of children with mostly normal systolic ventricular function, the large proportion of transplant recipients may limit generalizability of our findings. Future studies of WRF should focus on relatively large subpopulations with other physiologies including acute decompensated heart failure, functional single ventricles, and intracardiac mixing lesions. Second, the study design necessitated that we rely on non-standardized lab collection times. Presumably, sicker patients, some of whom may have greater degrees of venous congestion, underwent more frequent phlebotomy, possibly leading to bias in timing of WRF recognition. Similarly, our data have not captured additional renal stressors occurring after the initial catheterization such as the addition of nephrotoxic medications or additional procedures that may have contributed to WRF, and it’s possible these events would occur more frequently in those patients who are sicker to begin with. Third, our hemodynamic data were measured at only one point in time and therefore cannot reveal the influence of changing dynamics on renal function. Fourth, although we attempted to account for blood pressure in our analysis, blood pressure measurements vary widely within individuals during a cardiac catheterization and a single measurement is not sufficient for making associations and predictions. Ideally, changes in blood pressure would be analyzed instead. Finally, it is important to acknowledge that CVP measurements were made under general anesthesia and mechanical ventilation, and the influence of these conditions on venous hemodynamics in this cohort is uncertain.

## Conclusions

Among a cohort of children with a variety of cardiovascular disorders and mostly normal systolic ventricular function, higher CVP correlates with lower baseline eGFR and is independently associated with increased risk of WRF. Our data suggest that CVP, more so than cardiac index, is the main driver of renal function in children, although the effects of arterial blood pressure remain uncertain. Although static intracardiac hemodynamics likely make up a small proportion of all factors influencing baseline renal function, these data highlight the fundamental relationship between venous pressure and renal function and add to our understanding of the cardiorenal axis in children. More analysis of the longitudinal impact of CVP on renal function is warranted, and these observations highlight the need for further research into risk factors for WRF in children including those with heart failure and single ventricle physiology.

PERSPECTIVES **COMPETENCY IN MEDICAL KNOWLEDGE:** Despite widespread focus on cardiac output as a driver of renal dysfunction and injury, CVP is more consistently and significantly associated with renal function in children with cardiovascular disease.

**TRANSLATIONAL OUTLOOK:** Further investigation is needed to define the relationship between CVP and renal function in additional subpopulations of pediatric patients, and ultimately to explore the implications for management and renal outcomes particularly in the intensive care unit setting where continuous invasive pressure monitoring is available.

## Funding support and author disclosures

The authors have reported that they have no relationships relevant to the contents of this paper to disclose.
