# Supplementary material for: Central Venous Pressure and Impaired Renal Function in Children and Young Adults With Cardiovascular Disease
Source: JACC Adv. 2024 May 21;3(7):100995. doi: 10.1016/j.jacadv.2024.100995 (PMC11312305; doi:10.1016/j.jacadv.2024.100995)
Supplement: Supplemental Data [file mmc1.pdf]

Supplemental Table. Hemodynamic, demographic, and clinical associations with estimated glomerular filtration rate

Variable	Regression Coefficient (95% CI)	p-value	Multivariable Regression Coefficient (95% CI)	p-value
Age	-0.41 (-0.91, 0.10)	0.117		
Male sex	-4.0 (-10.67, 2.66)	0.238		
Transplant recipient	-6.51 (-13.1, 0.04)	0.051	-10.20 (-17.74, -2.65)	0.008
CVP	-2.34 (-3.21, -1.47)	<0.001	-2.65 (-4.02, -1.28)	<0.001
Cardiac index	0.82 (-1.98, 3.62)	0.566	-2.22 (-6.07, 1.64)	0.259
A-VO_2 difference	-0.34 (-0.70, 0.02)	0.061	-0.27 (-0.76, 0.23)	0.296
LV dysfunction	-12.34 (-23.32, -1.35)	0.028	-8.01 (-22.92, 6.90)	0.291
RV dysfunction	-11.37 (-22.20, -0.54)	0.040	-4.09 (-16.40, 8.21)	0.513
At least moderate TR	-8.10 (-19.26, 3.06)	0.154		
PCWP	-1.27 (-1.94, -0.60)	<0.001	-0.01 (-0.96, 0.97)	0.998
Mean PAP	-0.41 (-0.69, -0.13)	0.005		
Systolic BP z-score	0.02 (-0.01, 0.05)	0.194		
Diuretic	-2.90 (-10.65, 4.86)	0.462		
ACEi or ARB	1.49 (-5.74, 8.72)	0.685		
Beta blocker	-1.35 (-9.79, 7.09)	0.753		

ACEi=angiotensin converting enzyme inhibitor, ARB=angiotensin receptor blocker, A-VO_2=arterial-venous oxygen saturation, BP=blood pressure, CVP=central venous pressure, LV=left ventricular, PAP=pulmonary artery pressure, PCWP=pulmonary capillary wedge pressure, RV=right ventricular, TR=tricuspid regurgitation
